# Application of 3D Printed Models of Complex Hypertrophic Scars for Preoperative Evaluation and Surgical Planning

**DOI:** 10.3389/fbioe.2020.00115

**Published:** 2020-03-03

**Authors:** Peng Liu, Zhicheng Hu, Shaobin Huang, Peng Wang, Yunxian Dong, Pu Cheng, Hailin Xu, Bing Tang, Jiayuan Zhu

**Affiliations:** ^1^Department of Burn Surgery, The First Affiliated Hospital of Sun Yat-sen University, Guangzhou, China; ^2^Department of Burn and Plastic Surgery, Guangzhou Red Cross Hospital, Medical College, Jinan University, Guangzhou, China

**Keywords:** 3D printed models, hypertrophic scars, preoperative evaluation, surgical planning, wound scarring prevention

## Abstract

**Background:**

Complex hypertrophic scar is a condition that causes multiple joint contractures and deformities after trauma or burn injuries. Three-dimensional (3D) printing technology provides a new evaluation method for this condition. The objective of this study was to print individualized 3D models of complex hypertrophic scars and to assess the accuracy of these models.

**Methods:**

Twelve patients with complex hypertrophic scars were included in this study. Before surgery, each patient underwent a computed tomography (CT) scan to obtain cross-sectional information for 3D printing. Mimics software was used to process the CT data and create 3D printed models. The length, width, height, and volume measurements of the physical scars and 3D printed models were compared. Experienced surgeons used the 3D models to plan the operation and simulate the surgical procedure. The hypertrophic scar was completely removed for each patient and covered with skin autografts. The surgical time, bleeding, complications, and skin autograft take rate were recorded. All patients were followed up at 12 months. The surgeons, young doctors, medical students, and patients involved in the study completed questionnaires to assess the use of the 3D printed models.

**Results:**

The 3D models of the hypertrophic scars were printed successfully. The length, width, height, and volume measurements were significantly smaller for the 3D printed models than for the physical hypertrophic scars. Based on preoperative simulations with the 3D printed models, the surgeries were performed successfully and each hypertrophic scar was completely removed. The surgery time was shortened and the bleeding was decreased. On postoperative day 7, there were two cases of subcutaneous hemorrhage, one case of infection and one case of necrosis. On postoperative day 12, the average take rate of the skin autografts was 97.75%. At the 12-month follow-up, all patients were satisfied with the appearance and function.

**Conclusion:**

Accurate 3D printed models can help surgeons plan and perform successful operations, help young doctors and medical students learn surgical methods, and enhance patient comprehension and confidence in their surgeons.

## Introduction

Complex hypertrophic scar is a condition caused by trauma or burn injuries that may cause multiple joint contractures and deformities (Butzelaar et al., 2015; Anthonissen et al., 2016; Seo and Jung, 2016). The treatment of complex hypertrophic scars can dramatically improve a patient’s quality of life. At present, many conservative methods are used to treat complex hypertrophic scars; however, the outcomes are poor for patients with multiple skeletal deformities and scar contractures. Therefore, surgery is often the first treatment choice for patients with complex hypertrophic scars. When there are abnormal anatomical structures around the complex hypertrophic scar caused by skeletal deformities and soft tissue contractures, it is difficult for doctors to identify and assess the size of the scar clearly. Preoperative evaluation of complex hypertrophic scars is important for effective surgical treatment.

Currently, preoperative evaluation of complex hypertrophic scars depends exclusively on traditional, two-dimensional (2D) images, namely X-rays, computed tomography (CT), and magnetic resonance imaging (MRI). These types of imaging are used to evaluate the limits of complex hypertrophic scars and bone deformities; however, it is difficult to establish precise limits with 2D images (Ploch et al., 2016; Pfeil et al., 2017). Furthermore, it is also difficult to provide spatial anatomical information and tactile feedback for surgeons using these techniques.

Recently, 3D printing has been widely applied in orthopedic surgery, stomatology, and other medical fields because it has advantages in terms of individualization, tactility, and visualization (Cutroneo et al., 2016; Fitzhugh et al., 2016; Gu et al., 2016; Rashaan et al., 2016; Schepers et al., 2016). In this study, we made 3D models of complex hypertrophic scars to measure their dimensions preoperatively. We evaluated the accuracy of the 3D printed models. In addition, we assessed whether the 3D printed models were useful to surgeons in planning the operation, if they were helpful in the training of young doctors and medical students, and if they were useful tools for explaining the disease and operation to patients. Lastly, we assessed the clinical effect after surgery.

## Materials and Methods

### Patients

**FIGURE 1:**
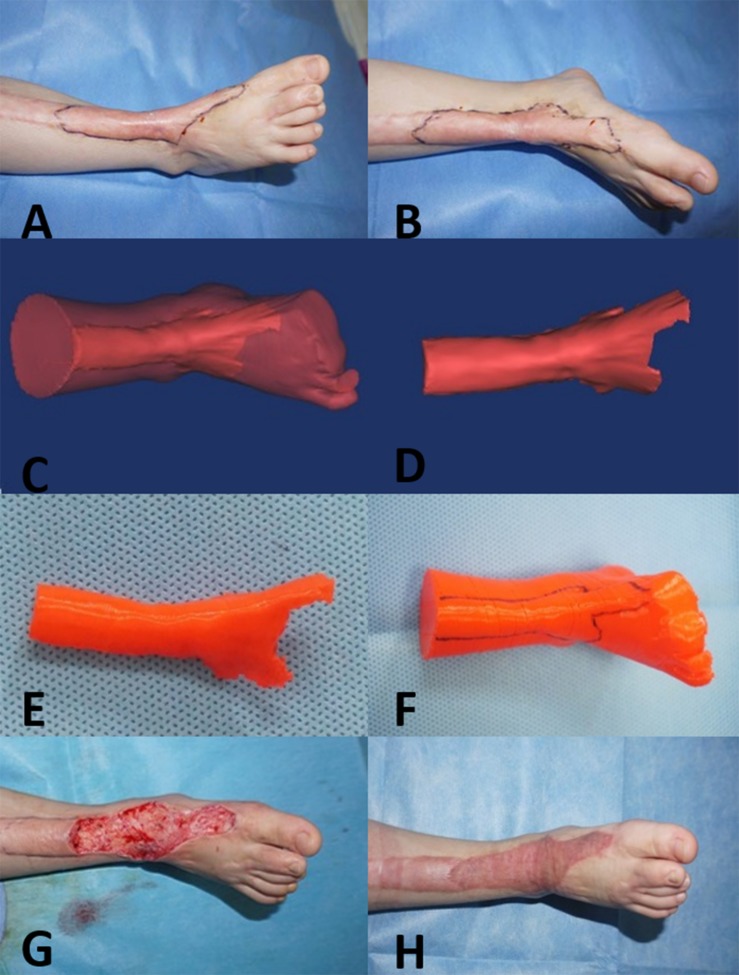
Three-year-old girl with a complex hypertrophic scar. **(A,B)** Preoperative images of the hypertrophic scar, which resulted in a loss of flexure and extension of the right ankle. **(C,D)** A 3D model of the hypertrophic scar was designed using reconstruction software. **(E,F)** The 3D model of the hypertrophic scar on the right ankle was printed for surgery simulation and anatomical measurement. **(G)** Based on the 3D printed model, the hypertrophic scar on the right ankle was completely removed surgically, and the wound was covered with razor-thin autologous skin. **(H)** At the 12-month follow-up visit, the appearance and function of right ankle were recovered.

**FIGURE 2:**
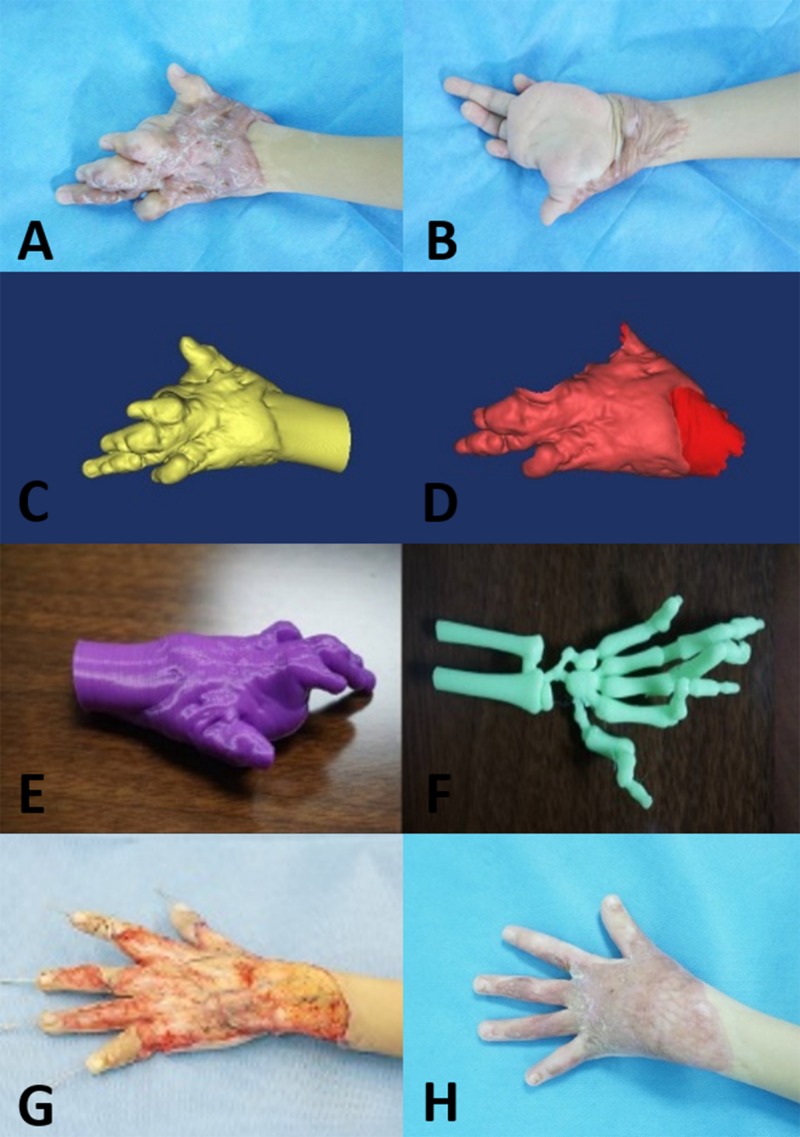
Five-year-old boy with a complex hypertrophic scar. **(A,B)** Preoperative images of the hypertrophic scar, which resulted in a loss of flexure and extension of the left hand. **(C,D)** A 3D model of the hypertrophic scar was designed using reconstruction software. **(E,F)** The 3D model of the hypertrophic scar on the left hand was printed for surgery simulation and anatomical measurement. **(G)** Based on the 3D printed model, the hypertrophic scar on the left hand was completely removed surgically, and the deformed bones were corrected with Kirschner wires. **(H)** At the 12-month follow-up visit, the appearance and function of the left hand were recovered.

Twelve patients who were hospitalized with complex hypertrophic scars from 1 December 2014 to 1 December 2015 were enrolled in this study. All patients experienced a loss of joint function and activity and exhibited severe deformity due to a complex hypertrophic scar (Figures 1, 2A,B). The study protocol was approved by the Institutional Review Board of The First Affiliated Hospital of Sun Yat-sen University, and informed consent was obtained from all participants.

### Image Processing and 3D Printing

**FIGURE 3:**
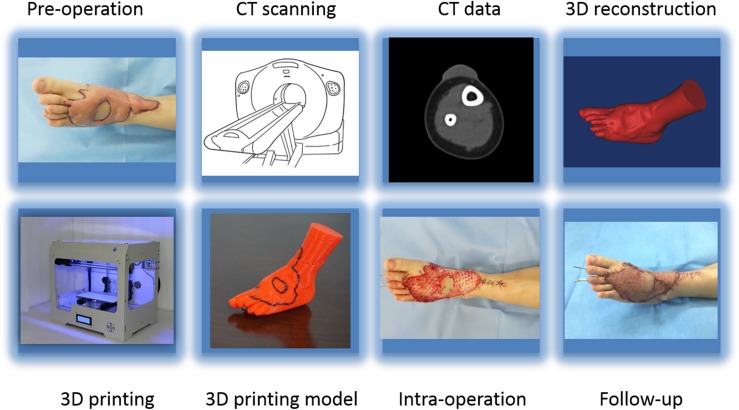
The workflow showing preoperation image acquisition, printing the 3D model, and follow-up.

A 64-slice spiral CT (Toshiba-Aquilion Corporation, Japan) was utilized to acquire serial cross-sectional data for each hypertrophic scar. Hypertrophic scar tissues were segmented from the optimal cross-sectional images with a thresholding tool using reconstruction software. Next, the 3D geometric models of the hypertrophic scar were exported as stereolithography (STL) format files for 3D printing (Figures 1, 2C,D). The STL format files were imported to PST-ZB (PST Photon Technology Co., Ltd., China), a rapid prototyping 3D printer with fused deposition modeling (FDM) principles. The printing material is polylactic acid (PLA), which is obtained by extracting starch from plants such as corn and cassava through multiple processes, fermenting it into lactic acid by microorganisms, and then polymerizing it. PLA is safer, lower in carbon, and greener compared with traditional materials. The printing parameters: printing speed 150 mm/s, temperature 200°C, and layer thickness 0.1 mm. The 3D scar models produced by the 3D printer were used preoperatively by experienced surgeons to simulate the surgical procedure to remove the hypertrophic scar. The printing process is shown in Figure 3.

### Validating Accuracy of the 3D Printed Models

**FIGURE 4:**
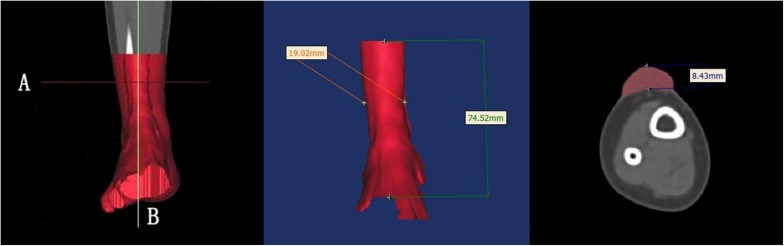
The length, width, and height of each hypertrophic scar were measured manually with rulers. These dimensions were measured with reconstruction software on the 3D printed models.

The length, width, and height of each hypertrophic scar were measured manually using rulers for the physical scar and with reconstructive software for the 3D printed model. Then the measurements of the physical scar and 3D printed model were compared as shown in Figure 4 (Olszewski et al., 2014; Yong et al., 2014; Lee et al., 2015; Wu et al., 2015; Yin et al., 2015). The volume of each 3D printed model was calculated automatically by Gemagic Quality software, and the volume of each 3D printed model was measured using the drainage method. These parameters were statistically analyzed by SPSS 13.0.

### Surgical Procedure and Postoperative Visits

Hypertrophic scar resection was performed for all 12 patients by the same group of experienced surgeons. Each hypertrophic scar was completely removed according to the measurement data and preoperative surgical simulation on the 3D printed models. A nurse recorded the surgery time and bleeding for each patient. Razor-thin skin autografts were harvested from the inner thigh to cover the scar area. The autografts were placed over human acellular dermal matrix scaffold (Jie-Ya Life Tissue Engineering, Beijing, China) intraoperatively and sutured to the graft area. Pressure was applied on the graft area. The 12 patients were followed up for 12 months after they were discharged from the hospital. At the 12-month follow-up visit, the skin autografts were assessed for skin color, appearance, elasticity, and texture at the suture.

### Evaluation of the 3D Models

The surgeons, young doctors, medical students, and patients evaluated the 3D printed models with specially designed feedback questionnaires. The responses to the questions were made on a 5-point Likert scale where 1 represents strongly disagree, 2 represents disagree, 3 represents neither agree nor disagree, 4 represents agree, and 5 represents strongly agree. The surgeons assessed the use of the models as surgical aids in terms of their visual appearance, quality, size, and surgical anatomy. The young doctors and medical students evaluated the use of the models for surgery simulation and training as well as the quality and size of the models. The patients assessed whether the use of the 3D printed models helped illustrate and explain the disease and helped them understand the surgical process and risks.

### Statistical Analysis

Statistical significance between groups was determined by paired *t*-test. All data were analyzed using SPSS version 16.0 software (IBM, Armonk, NY, United States).

## Results

Before surgery, individualized 3D models of the hypertrophic scars and deformed bones were successfully printed (Figures 1, 2E,F). The size and depth of the hypertrophic scar could be measured accurately on the 3D printed models. The average length, width, height, and volume of the physical hypertrophic scars and 3D printed models are presented in Table 1. The average length, width, and height of the 3D printed models were significantly smaller than the measurements of the physical scars. The average volume of the 3D scar models was significantly smaller than the average volume of the physical scars.

**TABLE 1:** Measurements of the patient scars and 3D printed models.

Parameter	Physical hypertrophic scar	3D printed model	*p*-value
Length (cm)	7.16 ± 2.17	7.10 ± 2.16	0.001*
Width (cm)	4.68 ± 1.40	4.57 ± 1.32	0.002*
Height (cm)	1.14 ± 0.37	1.10 ± 0.36	0.000*
Volume (ml)	38.22 ± 19.94	38.08 ± 19.94	0.001*

The maximum length, width, and height of the hypertrophic scar were measured with rulers and reconstruction software. The volume of the hypertrophic scar was measured using the water displacement method with a 5 L container. *n* = 12. *p < 0.05; paired *t*-test.

**FIGURE 5:**
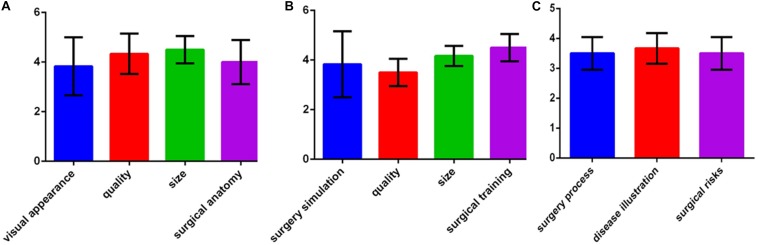
Assessments of the 3D printed models. **(A)** Evaluations by surgeons. **(B)** Evaluations by young doctors and medical students. **(C)** Evaluations by patients.

For each patient, surgery was completed according to the planned simulation by the same group of experienced surgeons, and the results were satisfactory. The medical students indicated that they had an improved comprehension of many surgical skills for resecting hypertrophic scars because of the simulated operations using the 3D printed models. The patients indicated that the explanations using the 3D printed models improved their understanding of the surgery and increased their trust of the surgeons. The average score of the evaluation about 3D printed models in each group on was greater than 3 points, which indicated that all of the groups were satisfied with the surgical simulations using the 3D printed models (Figure 5).

All patients successfully underwent hypertrophic scar resection according to the surgical simulations using the 3D printed models. The hypertrophic scar tissue was completely removed, and deformed bones were corrected according to the preoperative surgical plan (Figures 1, 2G). The surgical time was shortened and the bleeding was decreased. On postoperative day 7, there were two cases of subcutaneous hemorrhage, one case of infection and one case of necrosis, which may have been caused by excessive postoperative activity. On postoperative day 12, the average take rate of the skin autografts was 97.75% (Table 2). At the 12-month follow-up visit, all patients had satisfactory appearance and function (Figures 1, 2H).

**TABLE 2:** Patient characteristics and the take rate after surgery.

Patient	Sex	Age (years)	Cause of injury	Location	Injury time (years)	Take rate (%)*	Complication
1	Male	5	Hot water	Left hand	4	98	None
2	Female	19	Hot water	Left foot	10	97	Hematoma
3	Female	3	Flame	Right foot	2	99	None
4	Male	2	Hot water	Right hand	1	98	Hematoma
5	Male	16	Hot water	Left elbow joint	11	97	None
6	Female	4	Hot water	Right foot	2	98	Infection
7	Male	18	Hot water	Left hand	15	97	None
8	Female	3	Hot water	Right foot	2	97	Necrosis
9	Male	22	Flame	Left foot	14	98	None
10	Female	5	Hot water	Left hand	3	99	None
11	Female	26	Hot water	Left foot	18	97	None
12	Male	18	Flame	Right foot	9	99	None

*The take rates of skin autografts were recorded on postoperative day 12.

## Discussion

**FIGURE 6:**
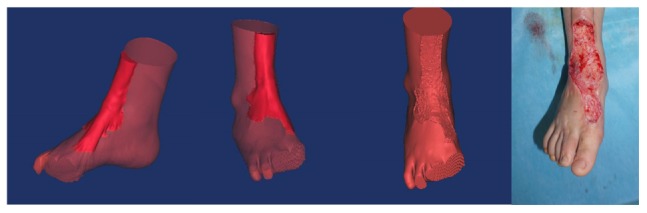
There is a clear limit between the hypertrophic scar and normal tissue. Surgeons should look for the surgical cutting plane to remove the integrative hypertrophic scar tissue.

Surgery is generally recommended for the treatment of hypertrophic scars. For surgery to be successful, it is important to identify the precise size of the hypertrophic scar (So et al., 2011; Amici, 2014; Lim et al., 2014; Orgill and Ogawa, 2014). The present study applied 3D printing to produce personalized models and observe the spatial position of the hypertrophic scar and bone deformity (Srougi et al., 2016). Using the models, anatomical measurements were made of the hypertrophic scar, including its length, width, and height (Silberstein et al., 2014). Our results suggest that 3D printed models of hypertrophic scars may guide surgeons to identify the surgical cutting plane that marks the limit between scar tissue and normal tissue. Knowledge of the surgical cutting plane can influence surgical effectiveness and potentially reduce complications (Figure 6).

Although 3D printing technology has been applied in many fields, it is necessary to evaluate its accuracy to meet clinical requirements. In the present study, the 3D printed models had a significantly smaller average length, width, height, and volume compared with the physical scars. These differences were caused by shrinkage of the material during printing, which affected the accuracy of the 3D models. The results of our study were similar to those of Lee et al. 2015; Wu et al., 2015). Although these differences were statistically significant, they were regarded as clinically insignificant. The 3D models served as valuable references for measuring anatomical parameters of the hypertrophic scar preoperatively, for planning the surgery, and for guiding the intraoperative manipulations.

The preoperative method for surgical resection of hypertrophic scar was direct measurement mainly to measure the size of hypertrophic scar, and the flap covered the wound after scar removal (Alali et al., 2015; Hwang et al., 2015; Martelli et al., 2016). However, this method lacked important parameters such as scar depth and volume, and cannot assess the anatomical relationship between scars and important anatomical structures such as nerves, blood vessels, and tendons, which in turn affects preoperative planning. Furthermore, we also successfully printed 3D models of deformed bones before surgery in the present study, measured the angle of the skeletal deformity, and corrected the angle of the deformity, which would be more helpful for surgeons to preoperative surgical evaluation and surgical planning (Figure 2F).

Many studies reported that 3D printed models were used in clinical practice and achieved good clinical results (Matsumoto et al., 2015; Rose et al., 2015; Powers et al., 2016; Youssef et al., 2016). Valverde et al. (2015) also used a Likert scale to assess the effect of 3D printed models by two experts for treatment of aortic hypoplasia and that it could reduce complications and operative time. Liu et al. (2014) demonstrated that the use of 3D printed models led to a 20% reduction in operating time. In this study, preparation of such 3D models for each hypertrophic scar patient can be feasible for surgeons. Preparation of 3D models has the following advantages. First, 3D printing can provide physiologically, anatomically, and tactilely realistic models before surgery. Second, individualized 3D models can be used for preoperative evaluation to reduce the operation time and bleeding, which can shorten hospital stay and reduce hospitalization costs. Third, individualized 3D models provide an effective way to improve communication and build trust between patients and doctors. Fourth, individualized 3D models may be used to simulate surgery and to teach new doctors (Alali et al., 2015; Hwang et al., 2015; Matsumoto et al., 2015; Rose et al., 2015; Martelli et al., 2016; Powers et al., 2016).

## Conclusion

Preoperative 3D printing technology can provide accurate 3D models to help surgeons plan operations to resect hypertrophic scars, help young doctors and medical students learn surgical methods, enhance communication and trust between patients and surgeons, and achieve good clinical effects.

## Data Availability Statement

All datasets generated for this study are included in the article/supplementary material.

## Ethics Statement

The studies involving human participants were reviewed and approved by the Institutional Review Board of  The First Affiliated Hospital of Sun Yat-sen University. Written informed consent to participate in this study was provided by the participants’ legal guardian/next of kin.

## Author Contributions

JZ and BT designed the research, and reviewed and edited the manuscript. PL, ZH, SH, BT, JZ, PW, YD, PC, and HX performed the experiment. PL, ZH, and SH wrote the manuscript. BT, JZ, ZH, and PL researched the data.

## Conflict of Interest

The authors declare that the research was conducted in the absence of any commercial or financial relationships that could be construed as a potential conflict of interest.
